# Beam steering for single antenna element using different optical lens

**DOI:** 10.1038/s41598-025-03751-9

**Published:** 2025-05-29

**Authors:** Aya W. Mohamed, Korany R. Mahmoud, Ahmed M. Montaser

**Affiliations:** 1 Electrical Engineering Department, Faculty of Technology and Education, Sohag University, PO. 82524, Sohag, Egypt; 2 Department of Electronics and Communications, Faculty of Engineering, Helwan University, PO. 11795, Cairo, Egypt

**Keywords:** Engineering, Electrical and electronic engineering

## Abstract

This paper presents a beam steering technique using optical lenses with a single antenna element, According to changing the illuminator element, the theoretical design of a hemispherical dielectric lens and an inhomogeneous dielectric flat lens modeled with various materials can increase and steer the radiation in a certain direction. The wavelength at which the nanoantenna functions is 1550 nm. The components of the plasmonic nanoantenna are silicon, silicon dioxide, and silver. The light inside the nanoantenna radiates vertically from the bottom to the top when it is lit from below. The gain of the nanoantenna is 8.46 dBi. The radiation efficiency rises when the lens is placed on top of the antenna because of the lens’s proportional construction. Two elements that significantly improve the antenna’s efficiency are the materials used to make the lens and the form of the lens. We chose two kinds of lenses: the flat lens, which is made up of 17 layers of various materials, and the hemispherical dielectric lens. To get the most gain and efficiency, the lenses are positioned above the nanoantenna. Additionally, the ability to steer the beam by moving the nanoantenna in accordance with various feeding points along the X and Z directions. Beam steering angles of ± 25° and ± 29° were attained by the hemispherical and flat lenses, respectively. Any lens can generally increase a nanoantenna’s efficiency, albeit different lenses offer varying degrees of efficiency and gain.

## Introduction

The authors in^1^ designed a plasmonic leaky wave nanoantenna using surface impedance modulation theory, which enables wide angle beam steering through a hybrid plasmonic waveguide with low loss. The authors designed elliptical plasmonic nanoantennas on a silicon substrate that can detect the vibrational fingerprint of polydimethylsiloxane (PDMS) for blood cancer diagnosis. These antennas can be adjusted based on doping concentration and structural dimensions^2^. The authors in^3^ designed an optical hybrid plasmonic patch nanoantenna that offers wide bandwidth, high gain, efficiency, directivity, and beam angle steering. The authors utilized a high-performance terahertz detector based on a two-dimensional plasmonic photoconductive nanoantenna array. This detector demonstrated improved sensitivity and wider bandwidth compared to non-plasmonic alternatives^4^. The authors in^5^ designed a graphene photodetector with plasmonic nanoantennas as electrodes, enabling concentrated carrier presence and broadband photodetection across visible and infrared wavelengths. This marks a significant demonstration of high responsivity over a wide bandwidth, made possible by plasmonic nanoantennas. The authors in^6^ designed a reflective array of plasmonic patch nanoantennas with an integrated indium tin oxide (ITO) layer for precise control of scanning angles and dynamic radiation patterns in the near infrared (NIR) range. In^7^, the authors used a hybrid plasmonic antenna for optical communications, suitable for various nanophotonic applications such as energy harvesting, sensing, and intra- and inter-chip communication, due to its broad frequency coverage and good gain. On the other hand, beam steering has been enabled at the nano- and micro-scale using magnetic fields to steer the radiation beams from plasmonic nanoantennas. Energy efficiency and integration in wireless communication are enhanced by reducing photonic elements^8^. On the other hand, a hybrid plasmonic nanopatch antenna with metal insulator metal (HMIM) multilayers offers good performance, such as gain, efficiency, and far field radiation. It is useful for many nanophotonic applications, such as beam steering^9^.

The authors in^10^ integrated a lens with a circularly polarized (CP) antenna made using low-cost 3D printing technology and a dielectric polarizer, employing a dielectric hemispherical lens to convert the antenna polarization from linear to circular. The CP lens can enhance the gain of the antenna. In^11^, the authors designed a loaded dielectric hemispherical lens antenna with five outputs to focus electromagnetic energy, enhance the gain, and achieve a wider bandwidth through the lens technique. The authors in^12^ designed a wideband antenna array that feeds a dielectric hemispherical lens, which is capable of simultaneously steering the beam to different positions while providing high gain. In^13^, the authors applied mathematical equations to design a hemispherical dielectric lens antenna that offers multifunctional operations. On the other hand, high gain can be achieved when a hemispherical dielectric lens is placed in the near field of the radiating elements^14^. The authors in^15^ were able to increase the gain of the quad-ridged horn antenna by combining it with a dielectric hemispherical lens. On the other hand, maximum directivity and radiation efficiency can be achieved when a logarithmic antenna is used on a hemispherical dielectric lens^16^. In^17^, the authors utilized phase center analysis to enhance the gain of lens antenna and reduce losses. There is a spacer between the lens and the top surface of the antenna to allow the signal to form a good phase front. In^18^, the authors used a lens to overcome scanning losses and achieve the highest gain when directing the beam. They created four different power splitters that direct the main beam at various angles. In^19^, the authors utilized a hemispherical dielectric lens with a printed log-periodic dipole array (PLPDA) antenna designed for operation in the V-band millimeter-wave frequency range to enhance both gain and radiation characteristics.

The authors in^20^ designed a flat dielectric fisheye lens that improved the aperture efficiency and bandwidth, allowing for better focus and direction of radiation and improved application performance. In^21^, the authors utilized a switched beam phased array system with a perforated flat Luneburg lens to enable effective and wide angle beam steering in millimeter-wave applications. The authors in^22^ designed a flat plasmonic lens based on gold that provides high transmission efficiency and ultrathin thickness. It is ideal for compact thermal imaging systems and optical integrated circuits. In^23^, the authors designed a flat Luneburg lens operating at 60 GHz, which enabled beam steering for wireless communication, featuring wide scanning angles and high gains. The authors in^24^ designed a single slab inhomogeneous zoned dielectric lens with independent phase correction capabilities, which offers improved performance, easier integration, and reduced cost, weight, and size compared to traditional lenses. In^25^, the authors designed ultrathin graphene-based Fresnel zone plate (FZP) lenses. These flat lenses offer advantages over conventional curved surface lenses, making them highly promising for compact optical systems and advanced electro-optical applications. The authors in^26^ employed a highly efficient terahertz gradient metasurface lens, enabling precise light control at a subwavelength scale. This facilitates ultrathin flat lenses with polarization insensitivity and efficient performance at wide angles, which holds great importance for imaging and communication applications. In^27^, the authors used a technique for flat lens design using field transformation (FT), surpassing ray optics (RO) and transformation optics (TO) methods. It offers broad bandwidth, higher field enhancement, and greater material flexibility without realizability issues. Precise control over phase and transmission makes it ideal for diverse optical and electromagnetic applications. On the other hand, a small circular metasurface lens is designed to reduce the main beam and thus enhance antenna gain^28^. In^29^, the authors designed a low-profile flat antenna with a wide aperture and the ability to direct the signal in the Ku band. It uses condensation index conversion material technology to operate with high efficiency and wide bandwidth, making it suitable for satellite communications and next-generation applications.

In our work, a beam steering technique will be presented using a hemispherical dielectric lens and a flat lens with a plasmonic nanoantenna at a frequency of 193.5 THz. The hemispherical dielectric lens and the flat lens are moved above the nano antenna until we achieve the highest gain. After that, the lenses are moved horizontally to the right and left to obtain the largest possible angles.

## Nanoantenna design

**Fig. 1:**
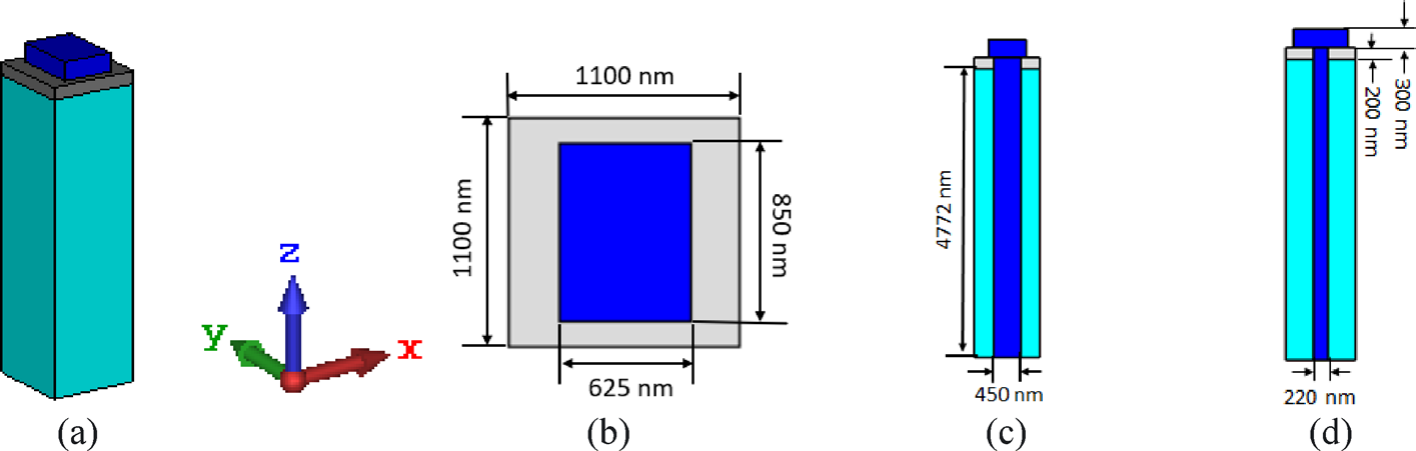
Nanoantenna fed by a silicon waveguide. (**a**) Perspective view, (**b**) top view, (**c**) front view, and (**d**) side view of nanoantenna^30^.

A nanoantenna is designed on the basis of a plasmonic structure and consists of silicon (Si), silicon dioxide (SiO_2_), and silver (Ag). The nanoantenna is fed by light from below by a silicon waveguide. The silicon waveguide travels through a mass of silver and is embedded within the silicon dioxide. The length of the silicon waveguide is 450 nm × 220 nm, the dimensions of the silver are 1100 nm × 1100 nm × 200 nm, the dimensions of silicon are 850 nm × 625 nm × 300 nm, and the dimensions of the silicon dioxide are 1100 nm × 1100 nm × 4772 nm. As shown in Fig. 1, the structure of the nanoantenna. The lower cutoff frequency of a mode in a rectangular waveguide is defined by the following equations:

1 $$\:{\left({f}_{c}\right)}_{mn}=\:\frac{c}{2}\sqrt{{\left(\frac{m}{a}\right)}^{2}+\:{\left(\frac{n}{b}\right)}^{2},}$$

where a and b are the waveguide’s width and length, respectively. The dominant mode in a waveguide is the one with the lowest cutoff frequency. ‘m’ and ‘n’ represent the different modes.

**Fig. 2:**
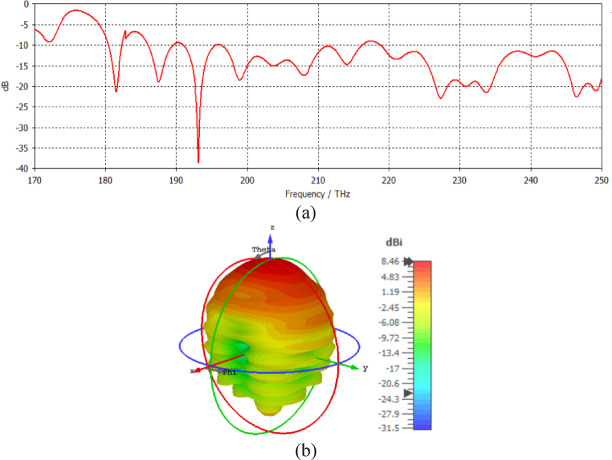
(**a**) Return loss and (**b**) 3D far field of the nanoantenna.

The CST-Microwave Studio program uses electromagnetic simulations to examine the radiation properties at 1550 nm. The relative permittivity of silicon is ε_r_ = 12.11, the relative permittivity of silicon dioxide is ε_r_ = 2.1, and the relative permittivity of silver is ε_r_ = − 129 + j3.28^17^. The loss tangent delta of silicon dioxide is 0.0005. The return loss at a frequency of 193.5 GHz is shown in Fig. 2a, and the 3D far field of the nanoantenna is shown in Fig. 2b.

## Discussion and results of hemispherical dielectric lens

A hemispherical dielectric lens is placed on top of the nanoantenna. Installation of a hemispherical dielectric lens with a low dielectric constant achieves high gain. The hemispherical dielectric lens is made of acrylic. The radius of the hemispherical dielectric lens is 2500 nm. The relative permittivity of acrylic is ε_r_ = 2.2.

Many researchers have used dielectric lenses in various applications. The Luneburg lens is a common spherical lens, but due to manufacturing challenges, spherical shells are often used as substitutes. Lenses serve two primary purposes in beam shaping and beam scanning. Primary lenses with specific shapes such as hyperbolic, elliptical, and hemispherical are used to collect the radiated energy with analytically derived parameters. However, it has been shown that Luneburg lenses do not provide optimal performance in terms of construction complexity^19^.

**Fig. 3:**
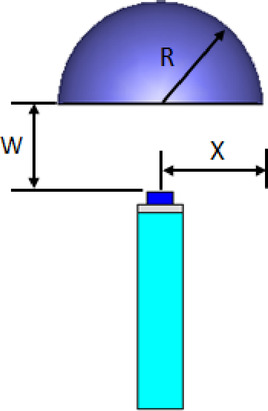
The nanoantenna with hemispherical dielectric lens.

**Fig. 4:**
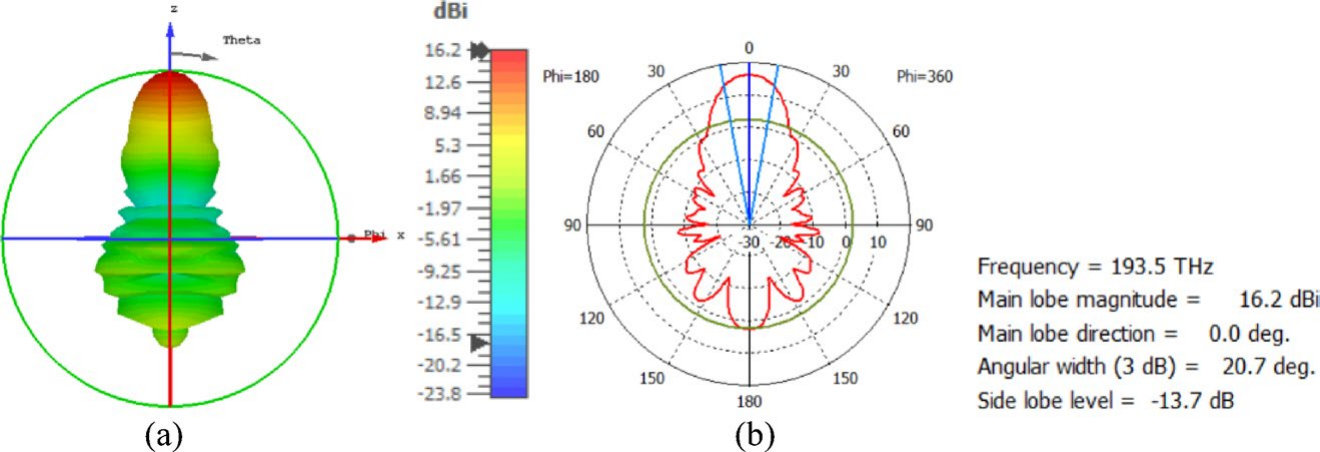
The distance between the lens and the antenna is W = 1000 nm vertically and its results (**a**) 3D far field, and (**b**) 2D polar plot radiation pattern.

**Fig. 5:**
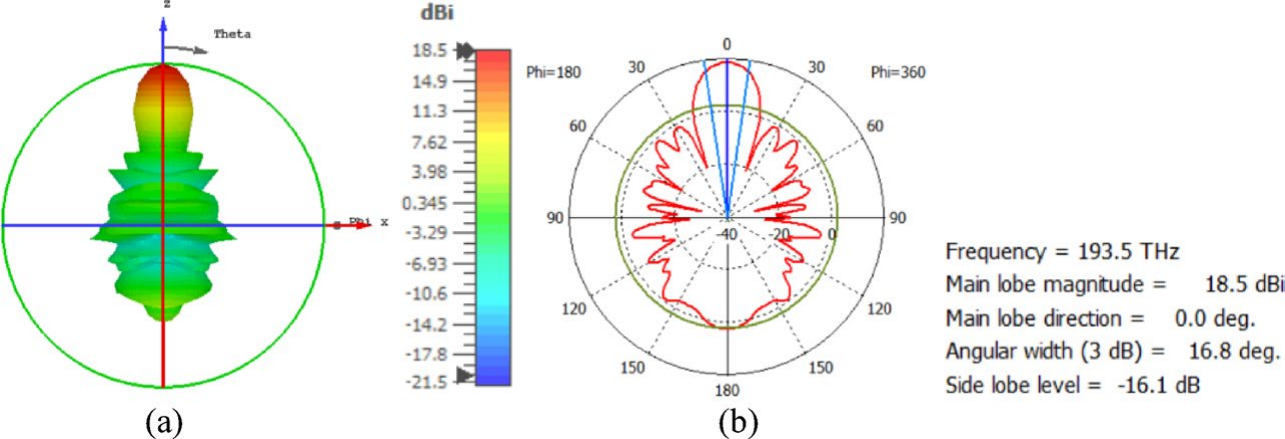
The distance between the lens and the antenna is W = 1900 nm vertically and its results (**a**) 3D far field, and (**b**) 2D polar plot radiation pattern.

**Fig. 6:**
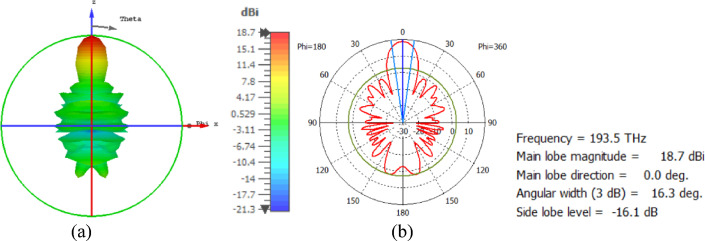
The distance between the lens and the antenna is W = 2128 nm vertically and its results (**a**) 3D far field, and (**b**) 2D polar plot radiation pattern.

**Fig. 7:**
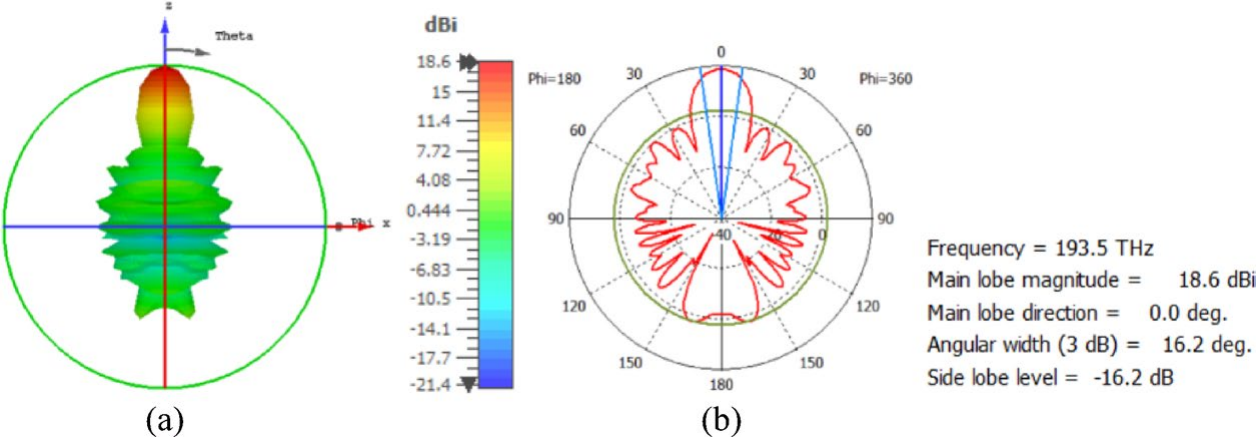
The distance between the lens and the antenna is W = 2200 nm vertically and its results (**a**) 3D far field, and (**b**) 2D polar plot radiation pattern.

**Fig. 8:**
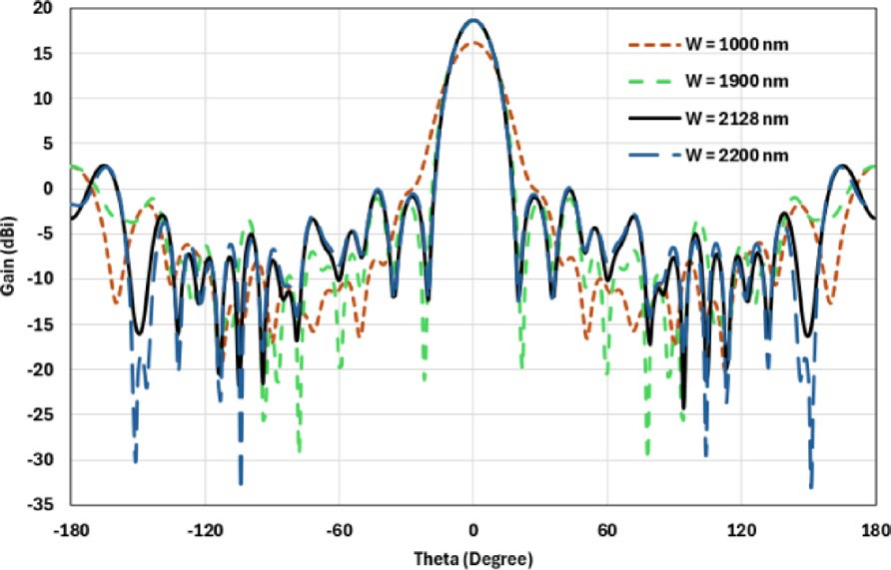
2D radiation pattern of vertical movements of a hemispherical dielectric lens above the antenna at φ = 180°.

The hemispherical dielectric lens is positioned at four different vertical locations above the nanoantenna. The parameters of the hemispherical lens include its radius (R) and the vertical distance (W) between the lens and the nanoantenna. The hemispherical dielectric lens is positioned above the nanoantenna, as shown in Fig. 3. The 3D far field of the hemispherical dielectric lens moves above the antenna in the z-axis direction at a distance of W = 1000 nm, with a gain of 16.2 dBi, as shown in Fig. 4a. The 2D polar plot of the radiation pattern is shown in Fig. 4b. Figure 5a shows the far field of the hemispherical dielectric lens moved above the antenna in the direction of the z-axis at a distance of 1900 nm, with a gain of 18.5 dBi. Figure 5b shows the 2D polar plot of the radiation pattern. The 3D radiation pattern of the hemispherical dielectric lens above the antenna when the distance between the lens and the antenna is W = 2128 nm and the gain is 18.7 dBi, as shown in Fig. 6a. The 2D polar plot of the radiation pattern is shown in Fig. 6b. Figure 7a shows the 3D far field when the vertical distance between the hemispherical dielectric lens and the antenna is 2200 nm and the gain is 18.6 dBi. Figure 7b shows the 2D polar plot of the radiation pattern. Figure 8 shows the 2D radiation pattern of vertical movements of a hemispherical dielectric lens above the antenna in the z plane (φ = 180°) and at a frequency of 193.5 THz. When we increase the distance between the lens and the antenna, we achieve better gain until we reach the optimal gain, which occurs at a distance of 2128 nm with a gain of 18.7 dBi. The distance related to the vertical movement of the hemispherical dielectric lens above the antenna, and gain, are listed in Table 1.

**Table 1:** List of the vertical distance between the hemispherical dielectric lens and the antenna, and gain.

The vertical distance between the lens and the antenna (nm)	Gain (dBi)
1000	16.2
1900	18.5
2128	18.7
2200	18.6

**Fig. 9:**
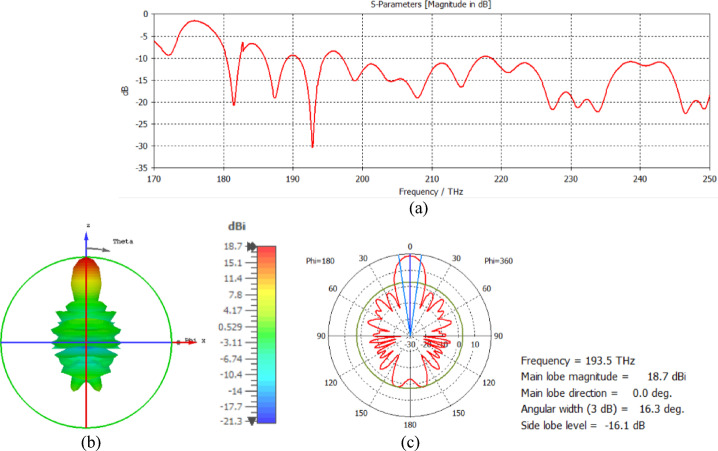
The distance between the lens and the antenna is X = 0 nm horizontally and its results (**a**) S_11_, (**b**) 3D far field, and (**c**) 2D polar plot radiation pattern at φ = 180°.

**Fig. 10:**
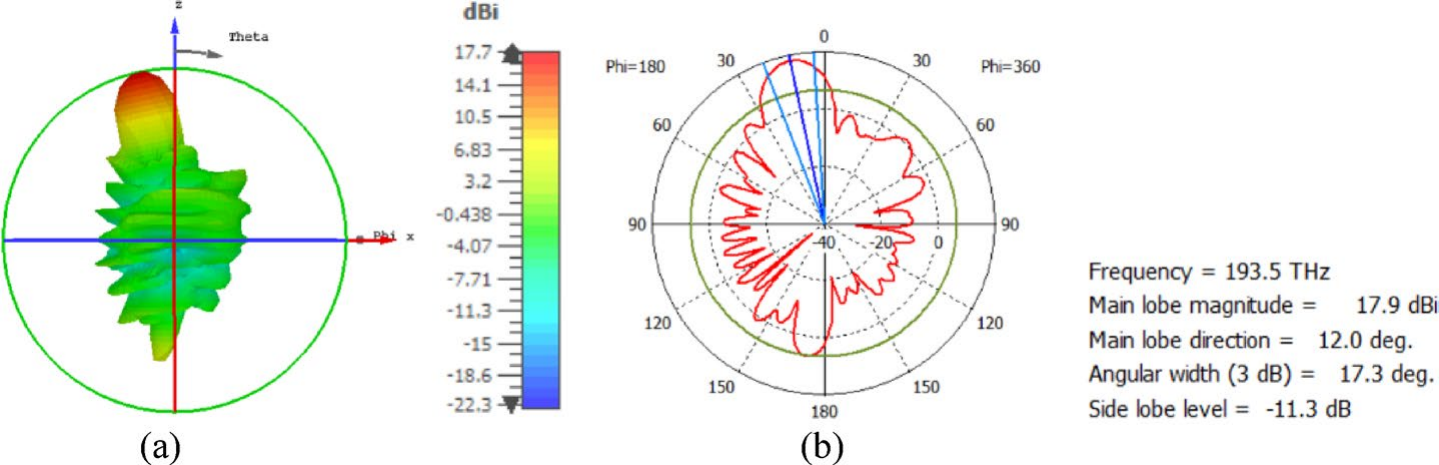
The distance between the lens and the antenna is X= − 900 nm horizontally and its results (**a**) 3D far field, and (**b**) 2D polar plot radiation pattern at φ = 180°.

**Fig. 11:**
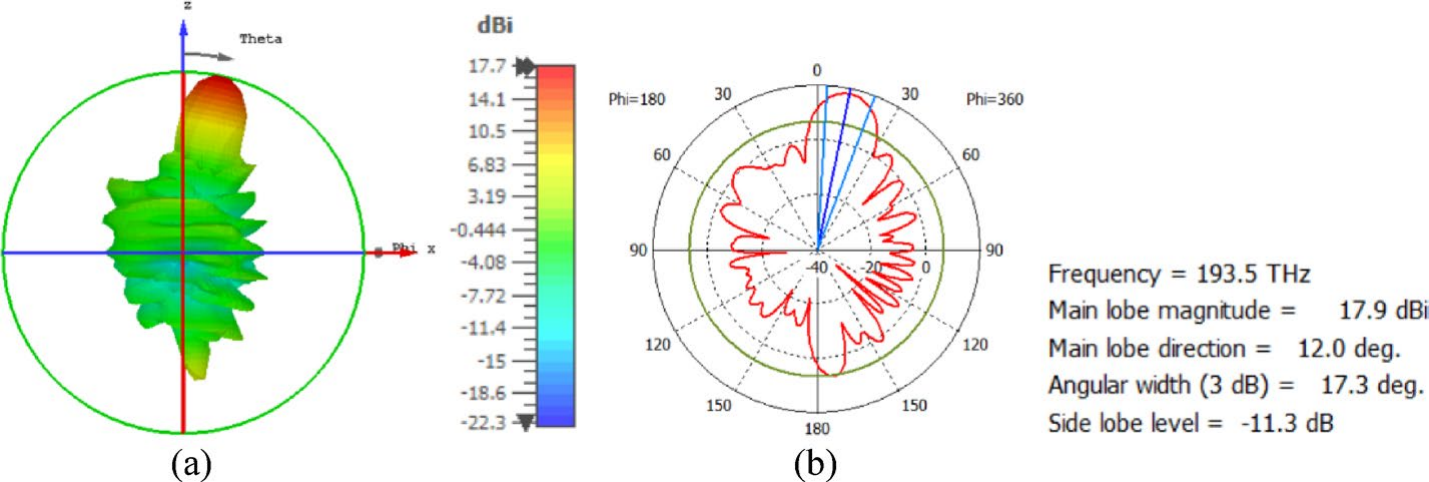
The distance between the lens and the antenna is X = 900 nm horizontally and its results (**a**) 3D far field, and (**b**) 2D polar plot radiation pattern at φ = 180°.

**Fig. 12:**
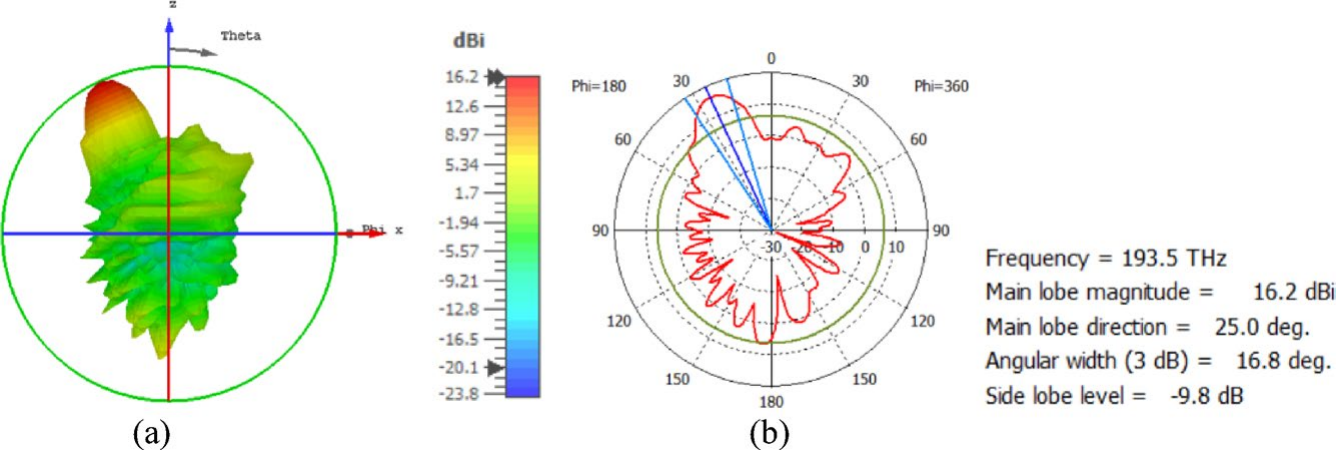
The distance between the lens and the antenna is X = − 1800 nm horizontally and its results (**a**) 3D far field, and (**b**) 2D polar plot radiation pattern at φ = 180°.

**Fig. 13:**
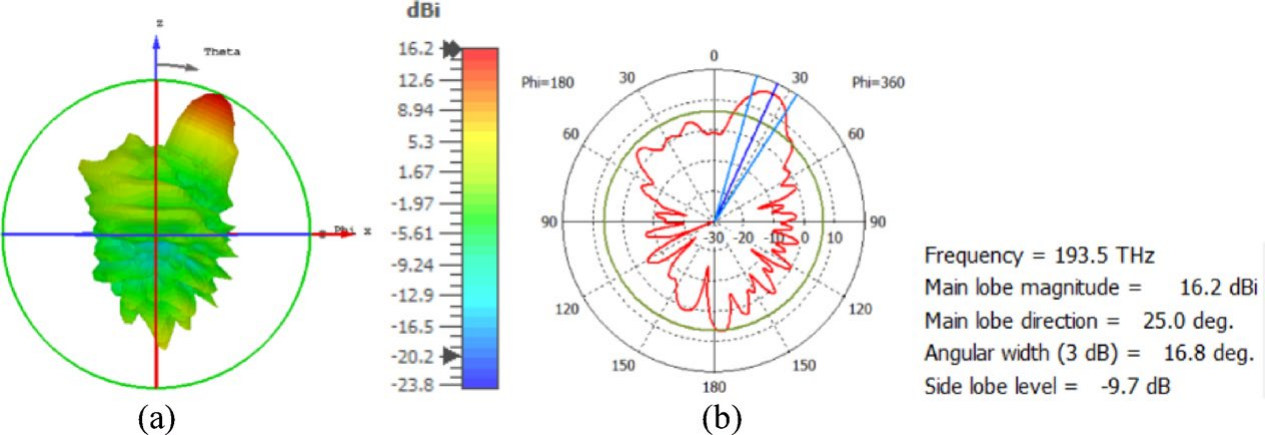
The distance between the lens and the antenna is X = 1800 nm horizontally and its results (**a**) 3D far field, and (**b**) 2D polar plot radiation pattern at φ = 180°.

**Fig. 14:**
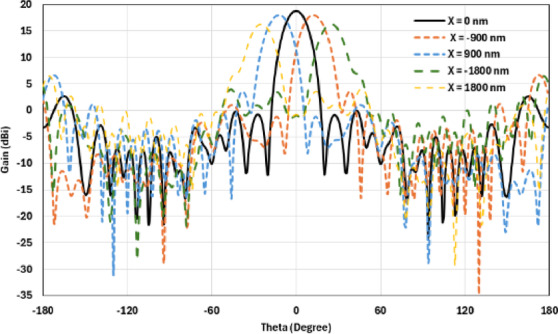
2D beam steering radiation pattern of horizontal movements of a hemispherical dielectric lens above the antenna at φ = 180°.

The hemispherical dielectric lens moves horizontally above the antenna in five positions, allowing us to direct the beam at different angles. The return loss of the antenna below the hemispherical dielectric lens is shown in Fig. 9a. The 3D radiation pattern of the antenna below the hemispherical dielectric lens, which has a gain of 18.7 dBi, is shown in Fig. 9b. The main lobe direction of the antenna below the hemispherical dielectric lens is 0°, as shown in Fig. 9c. The 3D far field of the hemispherical dielectric lens moves horizontally above the antenna at a distance of − 900 nm, with a gain of 17.7 dBi, as shown in Fig. 10a. The main lobe direction of the lens, positioned above the antenna at this distance of X = − 900 nm, is − 12°, as shown in Fig. 10b. Figure 11a shows the 3D far field radiation pattern of the hemispherical dielectric lens, which moves horizontally above the antenna in the direction of the x-axis at a distance of X = 900 nm, where the gain is 17.7 dBi. Figure 11b shows that the main lobe direction of the flat lens, moved horizontally above the antenna at a distance of X = 900 nm, is 12°. Figure 12a shows the 3D far field of the hemispherical dielectric lens moved over the antenna horizontally in the x-axis direction, with a distance of X = − 1800 nm and a gain is 16.2 dBi. Figure 12b the main lobe direction of the lens, moved horizontally above the antenna at a distance of X = − 1800 nm is − 25°. Figure 13a shows the 3D radiation pattern of the hemispherical dielectric lens moved above the antenna horizontally when the distance between the flat lens and the antenna is X = 1800 nm, with a gain of 16.2 dBi. Figure 13b shows the main lobe direction of the hemispherical dielectric lens, moved horizontally above the antenna at a distance of X = 1800 nm, is 25°. Figure 14 shows the 2D beam steering radiation pattern of horizontal movements of a hemispherical dielectric lens above the antenna in the x-z plane (φ = 180°) at a frequency of 193.5 THz, illustrating how the movement of the lens affects the radiation distribution. The horizontal distance between the center hemispherical dielectric lens and the center antenna, the main lobe direction, gain, directivity and Aperture Efficiency of lens are listed in Table 2.

**Table 2:** List of the horizontal distance between the center hemispherical dielectric lens and the center antenna, main lobe direction, gain, directivity and aperture efficiency of lens.

The horizontal distance between the center lens and the center antenna (nm)	Main lobe direction (°)	Gain (dBi)	Directivity (dBi)	Aperture efficiency of lens (%)
0	0	18.7	19.1	90.66
− 900	− 12	17.7	18.2	90.84
900	12	17.7	18.1	90.97
− 1800	− 25	16.2	16.7	90.8
1800	25	16.2	16.6	90.69

## Discussion and results of flat lens

**Fig. 15:**
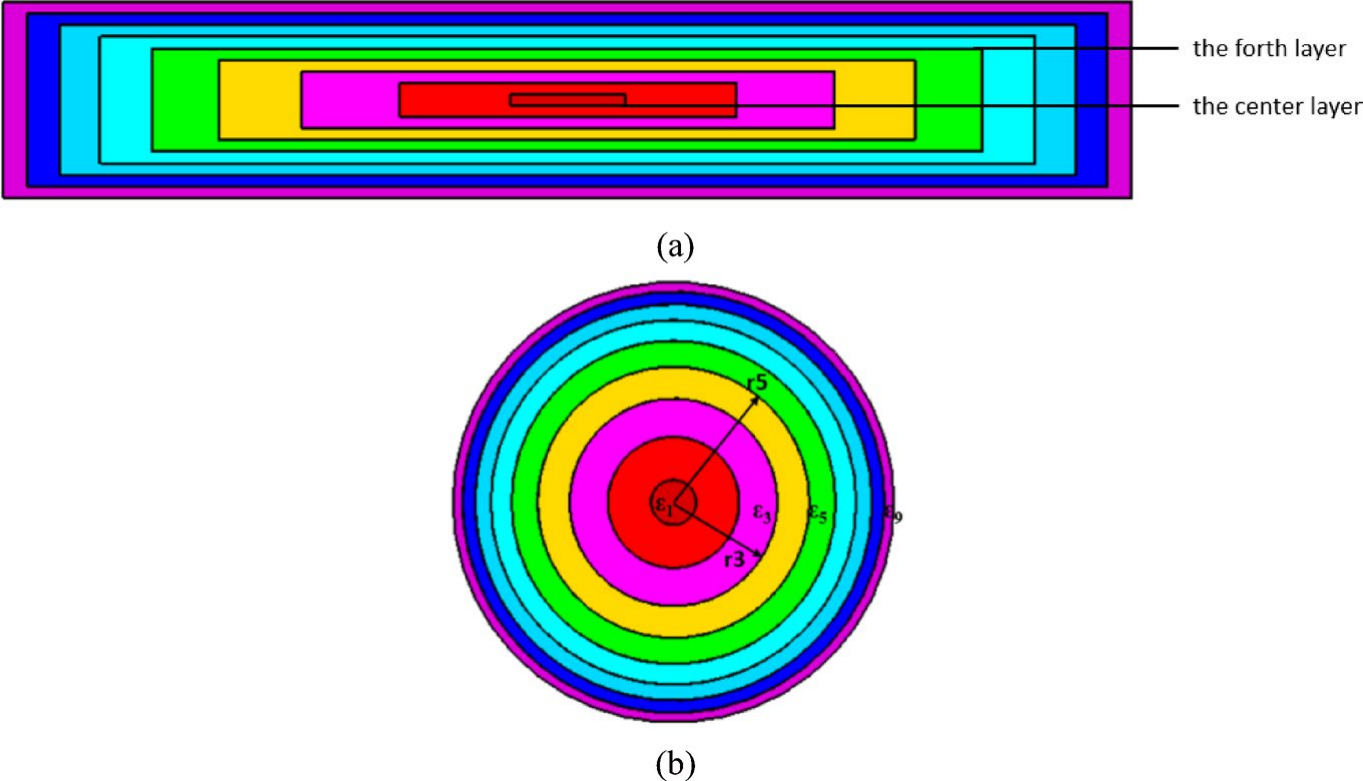
(**a**,** b**) Schematic of the flat lens composed by multilayer.

The flat lens is placed on top of the nanoantenna. When the flat lens is placed above the nanoantenna, it leads to achieving high gains. The lens has a diameter of 5100 nm and consists of 17 layers. Figure 15a and b show the structure of the flat lens made of multi-layer. The design of the flat lens profile can be determined using Eqs. (2) and (3)^31^. The radius (Ri) for each dielectric zone can be determined using Eq. (2):

2 $$\:{R}_{i}=\sqrt{2Fi\left(\frac{\lambda\:}{p}\right)+{\left(i\frac{\lambda\:}{p}\right)}^{2},\:\:}\:i=2,\:3,\:.\:.\:.,p,$$

where P is the phase correcting index is equal to 9, λ is the design wavelength and F is the focal length. The thickness of the lens H is related to the two adjacent permittivities of the lens and it can be obtained by using Eq. (3):

3 $$\:H=\frac{\lambda\:}{p\left(\sqrt{{{\upepsilon\:}}_{ri}\:}-\:\sqrt{{{\upepsilon\:}}_{ri}-1}\right)},\:i=2,\:3,\:\dots\:,p.$$

The focal-to-diameter (f/D) of the lens is about 0.5. The dimensions of the radius of the cylinder for all layers are listed in Table 3. The lens is symmetrical around L0, and the lens consists of 9 permittivity zones. The thickness of each layer is 52.04 nm. The permittivity distributions for each layer are ε_r1_ = 10, ε_r2_ = 9.13, ε_r3_ = 7.86, ε_r4_ = 6.45, ε_r5_ = 5.13, ε_r6_ = 4.00, ε_r7_ = 3.15, ε_r8_ = 2.49, ε_r9_ = 2.01.

**Table 3:** The radius of the cylinder for all layers.

Layer	*r*_1_	*r*_2_	*r*_3_	*r*_4_	*r*_5_	*r*_6_	*r*_7_	*r*_8_	*r*_9_
L_0_	260.2	759.79	1202.14	1571.63	1873.46	2112.85	2295.57	2440.71	2550
L_1_		759.79	1202.14	1571.63	1873.46	2112.85	2295.57	2440.71	2550
L_2_			1202.14	1571.63	1873.46	2112.85	2295.57	2440.71	2550
L_3_				1571.63	1873.46	2112.85	2295.57	2440.71	2550
L_4_					1873.46	2112.85	2295.57	2440.71	2550
L_5_						2112.85	2295.57	2440.71	2550
L_6_							2295.57	2440.71	2550
L_7_								2440.71	2550
L_8_									2550

**Fig. 16:**
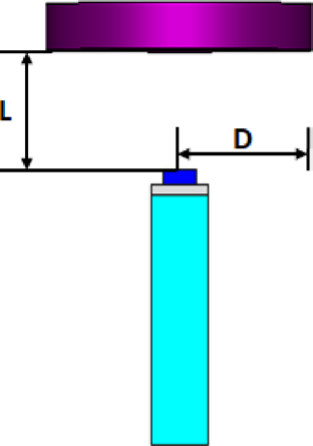
The nanoantenna with flat lens.

**Fig. 17:**
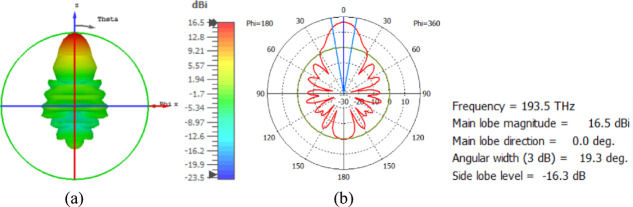
The distance between the lens and the antenna is L = 1583 nm vertically and its results (**a**) 3D far field, and (**b**) 2D polar plot radiation pattern.

**Fig. 18:**
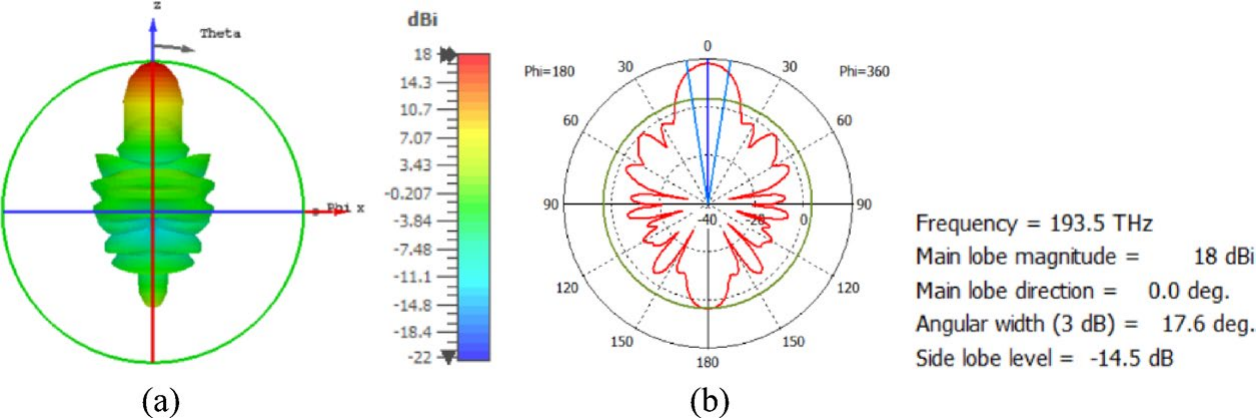
The distance between the lens and the antenna is L = 2083 nm vertically and its results (**a**) 3D far field, and (**b**) 2D polar plot radiation pattern.

**Fig. 19:**
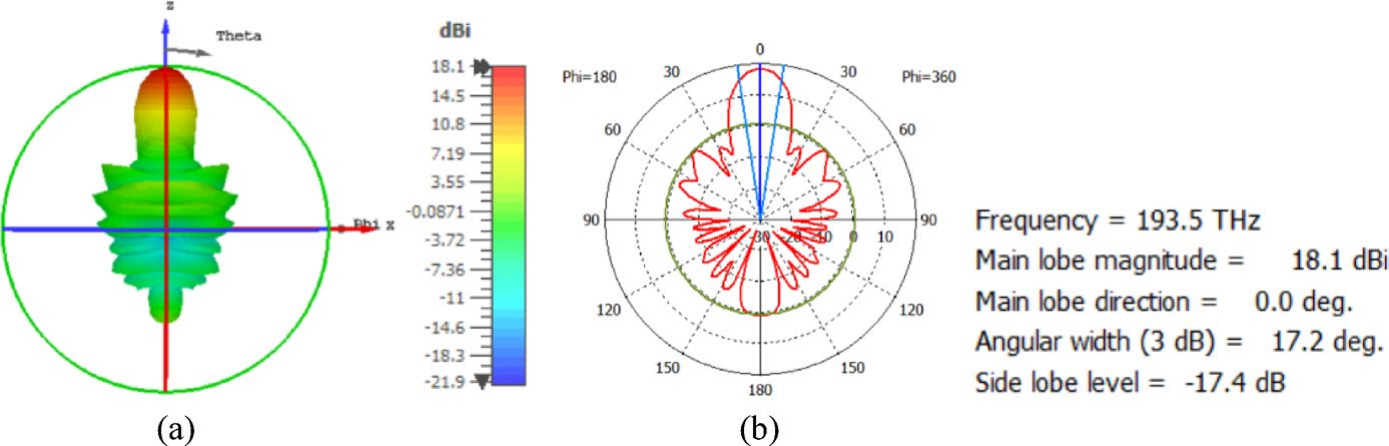
The distance between the lens and the antenna is L = 2283 nm vertically and its results (**a**) 3D far field, and (**b**) 2D polar plot radiation pattern.

**Fig. 20:**
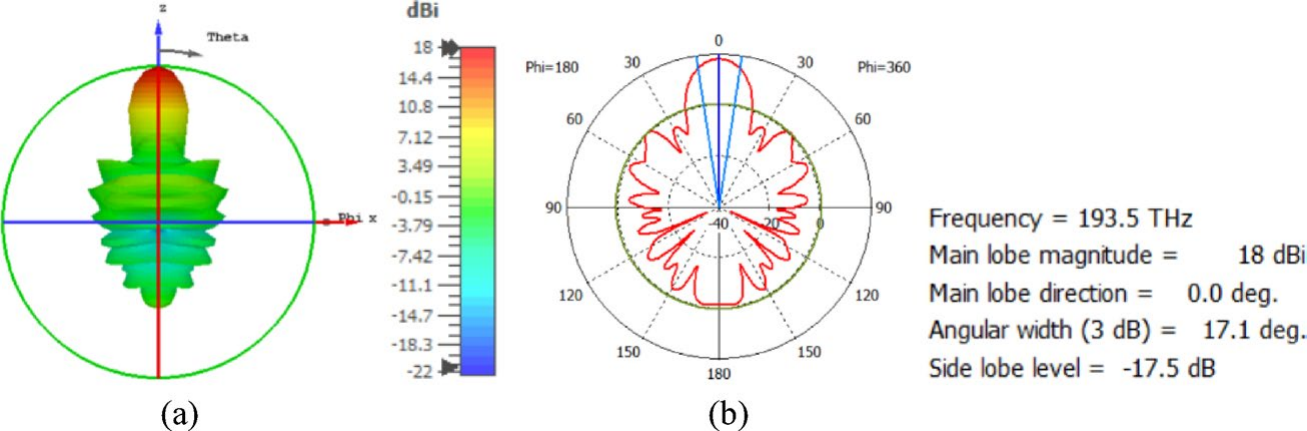
The distance between the lens and the antenna is L = 2383 nm vertically and its results (**a**) 3D far field, and (**b**) 2D polar plot radiation pattern.

**Fig. 21:**
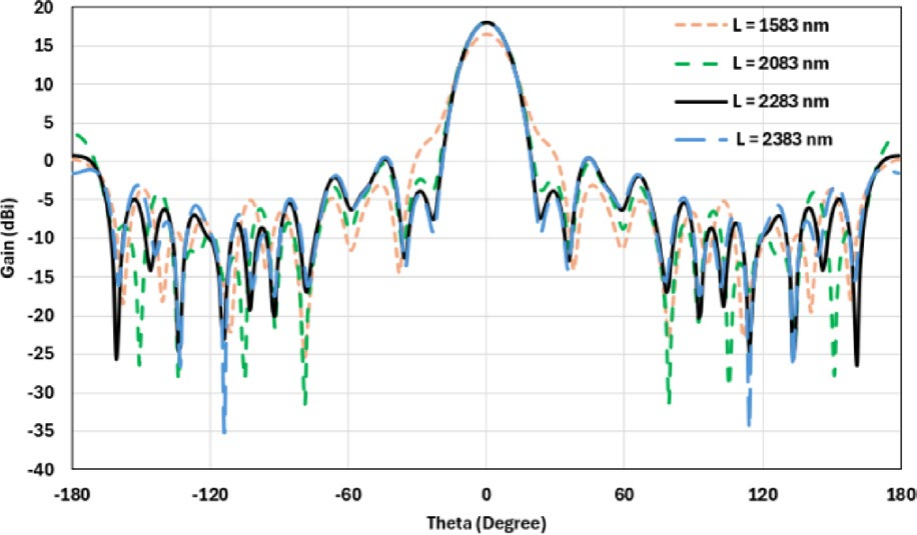
2D radiation pattern of vertical movements of a flat lens above the antenna at φ = 180°.

The flat lens is placed at four different vertical positions above the nanoantenna, where L indicates the specific vertical distance from the lens to the nanoantenna. The flat lens is positioned above the nanoantenna, illustrating how the flat lens moves in the L and D directions, as shown in Fig. 16. Figure 17a shows the far field radiation pattern when the vertical distance between the antenna and the flat lens is L = 1583 nm, with a gain of 16.5 dBi. Figure 17b shows the 2D polar plot radiation pattern. Figure 18a shows the 3D far field of the flat lens moved over the antenna vertically in the z-axis direction, with distance between the flat lens and the antenna is L = 2083 nm, and the gain is 18 dBi. Figure 18b shows the 2D polar plot radiation pattern. Figure 19a shows the 3D radiation pattern of the flat lens moved vertically above the antenna when the distance between the flat lens and the antenna is L = 2283 nm, with a gain of 18.1 dBi. Figure 19b shows the 2D polar plot of the radiation pattern. The 3D far field of the flat lens above the antenna when the distance between the lens and the antenna is L = 2383 nm and the gain is 18 dBi, as shown in Fig. 20a. The 2D polar plot of the radiation pattern is shown in Fig. 20b. Figure 21 shows the 2D radiation pattern of vertical movements of a flat lens above the antenna in the z plane (φ = 180°) and at a frequency of 193.5 THz. When we increase the distance between the flat lens and the antenna, we achieve better gain until we reach the optimal gain, which occurs at a distance of 2283 nm with a gain of 18.1 dBi. The distance related to the vertical movement of the flat lens above the antenna, and gain, are listed in Table 4.

**Table 4:** List of the vertical distance between the flat lens and the antenna, and gain.

The vertical distance between the lens and the antenna (nm)	Gain (dBi)
1583	16.5
2083	18
2283	18.1
2383	18

**Fig. 22:**
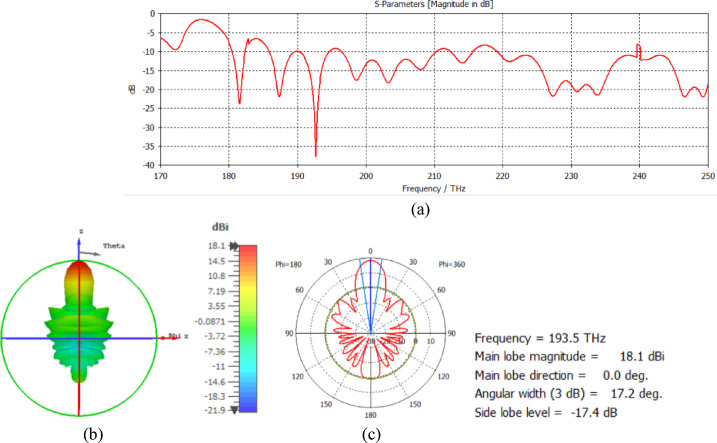
The distance between the lens and the antenna is D = 0 nm horizontally and its results (**a**) S_11_, (**b**) 3D far field, and (**c**) 2D polar plot radiation pattern at φ = 180°.

**Fig. 23:**
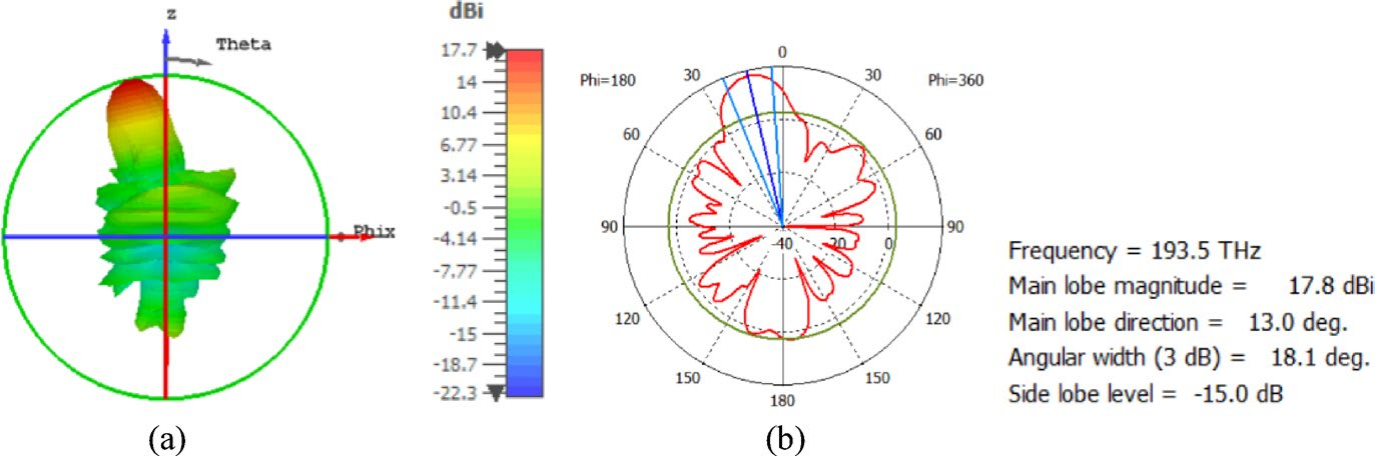
The distance between the lens and the antenna is D = − 900 nm horizontally and its results (**a**) 3D far field, and (**b**) 2D polar plot radiation pattern at φ = 180°.

**Fig. 24:**
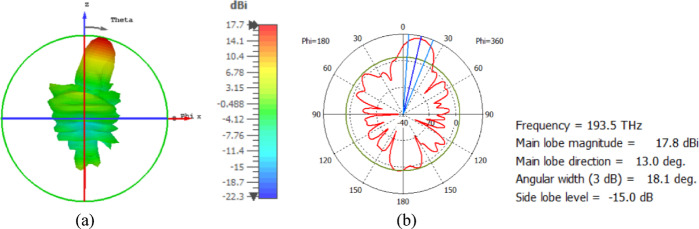
The distance between the lens and the antenna is D = 900 nm horizontally and its results (**a**) 3D far field, and (**b**) 2D polar plot radiation pattern at φ = 180°.

**Fig. 25:**
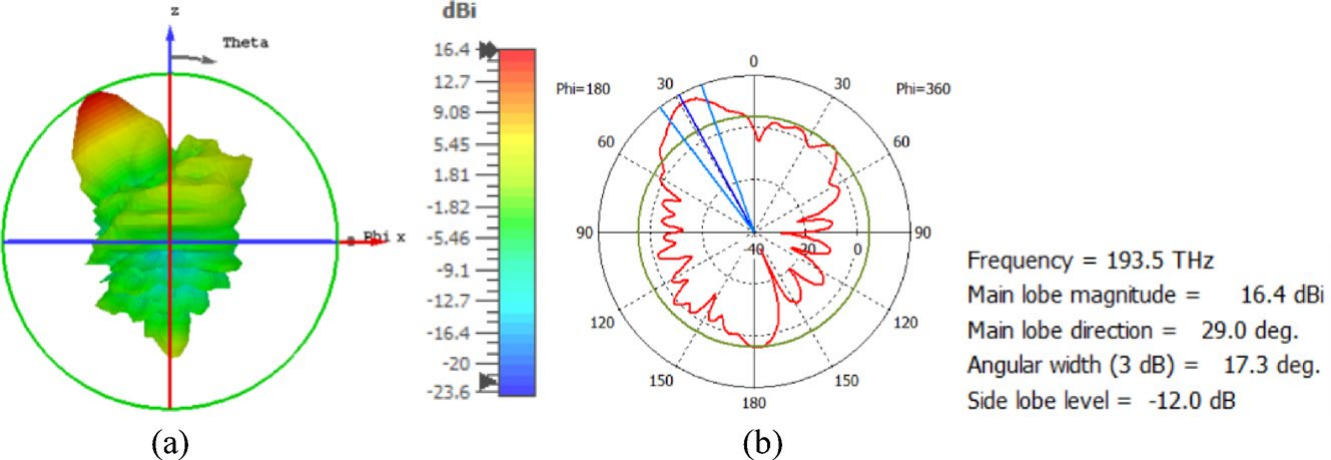
The distance between the lens and the antenna is D = − 1900 nm horizontally and its results (**a**) 3D far field, and (**b**) 2D polar plot radiation pattern at φ = 180°.

**Fig. 26:**
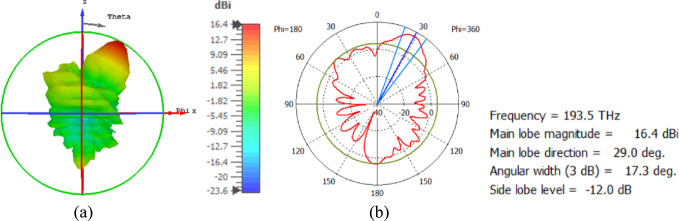
The distance between the lens and the antenna is D = 1900 nm horizontally and its results (**a**) 3D far field, and (**b**) 2D polar plot radiation pattern at φ = 180°.

**Fig. 27:**
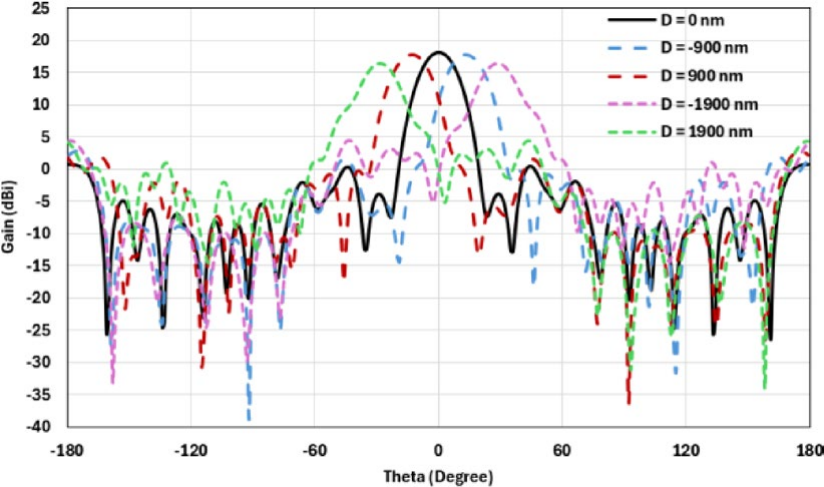
2D beam steering radiation pattern of horizontal movements of a flat lens above the antenna at φ = 180°.

The flat lens moves horizontally above the antenna in five positions, and we get to direct the beam at different angles. The return loss of the flat lens above the antenna is shown in Fig. 22a. The 3D far field radiation pattern of the antenna below the flat lens and the gain is 18.1 dBi, as shown in Fig. 22b. The main lobe direction of the antenna below the flat lens is 0°, as shown in Fig. 22c. Figure 23a shows the 3D radiation pattern of the flat lens moved horizontally above the antenna at a distance of D = − 900 nm, with a gain of 17.7 dBi. Figure 23b shows that the main lobe direction of the flat lens moved horizontally above the antenna at a distance of D = − 900 nm is − 13°. The 3D far field of the flat lens moved horizontally above the antenna at a distance equal to 900 nm, and the gain is 17.7 dBi, as shown in Fig. 24a. The main lobe direction of the lens moved above the antenna horizontally at a distance of D = 900 nm is 13°, as shown in Fig. 24b. Figure 25a shows the 3D far field radiation pattern of the flat lens which moves horizontally above the antenna in the direction of the x-axis at a distance of D = − 1900 nm, where the gain is 16.4 dBi. Figure 25b shows that the main lobe direction of the flat lens moved horizontally above the antenna at a distance of D = − 1900 nm is − 29°. The 3D far field of the flat lens moved horizontally above the antenna at a distance of D = 1900 nm, with a gain of 16.4 dBi, as shown in Fig. 26a. The main lobe direction of the flat lens moved horizontally above the antenna at a distance of D = 1900 nm is 29°, as shown in Fig. 26b. Figure 27 shows the 2D beam steering radiation pattern of horizontal movements of a flat lens above the antenna in the x-z plane (φ = 180°) and at a frequency of 193.5 THz, illustrating how the movement of the lens affects the radiation distribution. The horizontal distance between the center flat lens and the center antenna, the main lobe direction, gain, directivity and Aperture Efficiency of lens are listed in Table 5.

**Table 5:** List of the horizontal distance between the center flat lens and the center antenna, main lobe direction, gain, directivity and aperture efficiency of lens.

The horizontal distance between the center lens and the center antenna (nm)	Main lobe direction (°)	Gain (dBi)	Directivity (dBi)	Aperture efficiency of lens (%)
0	0	18.1	18.5	91.14
− 900	− 13	17.7	18	92.75
900	13	17.7	18	92.85
− 1900	− 29	16.4	16.7	91.65
1900	29	16.4	16.7	91.67

The placement of a lens on the antenna results in beam forming. The lens focuses and shapes the electromagnetic waves emitted by the antenna, directing them towards specific points or areas of interest with increased precision. This concentration of the signal enhances signal strength and improves the signal-to-noise ratio, leading to better communication performance and increased range. In the case of moving the hemispherical dielectric lens and the flat lens to the right and left in the X direction at different distances, various angles and gains achieved are listed in Table 6.

The aperture efficiency of a beam steering lens antenna is a measure of how effectively the lens concentrates the electromagnetic energy emitted from the source into a narrow, directed beam. This efficiency is influenced by several factors, including the design of the lens, such as its shape and the material from which it is made, as well as the characteristics of the signal source, including the signal frequency and polarization, and the size of the aperture. Aperture efficiency is typically measured as the ratio of the actual energy radiated in the main beam to the total energy that the aperture radiates. It is an important criterion for evaluating the performance of a beam steering lens antenna, as it directly affects the antenna’s gain and side lobe width, influencing communication quality and the antenna’s ability to resist interference.

In this section, a lens is used to accomplish 2D beam steering without having to tune the wavelength. Compared to the two lenses, the hemispherical lens operated the beam steering range from − 25° to 25° in addition to keeping the gain value approximately uniform at 18.7 dBi at 0° and changing at ± 25° with a gain of 16.2 dBi. Then, the beam steering is achieved by switching between the feed antenna elements, whereas the feed antenna array is attached to the back surface of the lens collimator. The flat lens antenna system achieved 2D beam steering capability, whereas with a lens with a maximum measured directivity of 18.1 dBi, an uninterrupted beam-switching range of around ± 29° is established.

**Table 6:** Comparison between the results of a hemispherical dielectric lens and a flat lens at φ = 180°.

The hemispherical dielectric lens			The flat lens		
The horizontal distance between the center lens and the center antenna (nm)	Main lobe direction (°)	Gain (dBi)	The horizontal distance between the center lens and the center antenna (nm)	Main lobe direction (°)	Gain (dBi)
0	0	18.7	0	0	18.1
− 900	− 12	17.7	− 900	− 13	17.7
900	12	17.7	900	13	17.7
− 1800	− 25	16.2	− 1900	− 29	16.4
1800	25	16.2	1900	29	16.4

Our work has been compared with literary references to verify its quality against other research. It was found that our work meets the required specifications and exceeds many existing studies, as shown in Table 7.

**Table 7:** A comparison of other studies and our work.

Performance	Ref.^32^	Ref.^33^	Ref.^34^	Ref.^35^	Ref.^35^	Ref.^36^	Ref.^37^	Our work	
								The flat lens	The hem. lens
Dimension (mm)	104 × 108	60 × 60 × 50.76	228.6	95.4 × 95.4 × 90	95.9 × 95.9 × 95.9	98.9 × 98.9 × 44.8	7.75 × 10^− 3^ × 2.17 × 10^− 3^	5.1 × 10^− 3^ × 8.85 × 10^− 4^	5 × 10^− 3^
Frequency (GHz)	12.4:18	26:40	8:12	10	10	9, 10, 11	193,500	193,500	193,500
Max. gain (dBi)	23.7	23.5	~ 27 dB (Dir.)	16.8 dBi	18.3 dBi (Dir.)	15.88 (H) 16.35 (V)	18.4	18.1	18.7
Scanning angle (°)	± 50	± 55	± 15	± 34	± 90	± 32 (H) ± 35 (V)	± 60 ± 55	± 29	± 25
BW of gain flatness (GHz)	5.6	14	4	> 2	> 4	~ 2	11,000	24,400	30,900
Back radiation (dB)	− 16.3	− 42.8	N.A.	− 13	< − 13	~ − 10	6.1	− 17.4	− 16.1
Aperture efficiency (%)	N.A.	> 60	N.A.	~ 67	52.6	46.2	N.A.	91.14	90.66

In recent years, the manufacturing of lens antennas has increasingly relied on advanced techniques, especially 3D printing, which offers a precise and cost-effective way to create complex geometries required for high-performance lenses. 3D printing enables rapid prototyping and customization of lens designs, which can be difficult to achieve with traditional machining methods. These additive manufacturing processes, such as stereolithography (SLA)^38^ or selective laser sintering (SLS)^39^, allow for the fabrication of lenses with unique shapes, fine features, and graded-index structures that can enhance antenna performance. After manufacturing, the lenses are typically validated through various measurements, including gain, beamwidth, and efficiency tests. Measured results often show close alignment with simulated performance, confirming the accuracy and reliability of 3D-printed lenses. This approach has proven successful in applications from millimeter-wave systems to terahertz frequencies, where precise wavefront control is essential.

## Conclusion

This paper presents a comparison between two lenses: the hemispherical dielectric lens with a plasmonic nanoantenna and the flat lens with a plasmonic nanoantenna. The plasmonic nanoantenna operates at a frequency of 193.5 THz, achieving a gain of 8.46 dBi. Both lenses performed well. The hemispherical dielectric lens with a radius of 2500 nm achieved a gain of 18.7 dBi and beam steering angles of ± 25°, while the flat lens with a radius of 2550 nm, which consists of 17 layers made of different materials, achieved a gain of 18.1 dBi and beam steering angles of ± 29°. Each lens is utilized according to the appropriate application, such as optical communications, sensing technologies, medical imaging, and remote sensing systems.

## Data Availability

The datasets used and/or analyzed during the current study available from the corresponding author on reasonable request.
